# Preferences for Nonpharmaceutical Interventions During the Endemic Phase of COVID-19: Discrete Choice Experiment

**DOI:** 10.2196/67725

**Published:** 2025-06-04

**Authors:** Yi Wang, Chee Ern Har, Sharon Hui Xuan Tan, Hooi Swang Cheng, Ian Yi Han Ang

**Affiliations:** 1 Saw Swee Hock School of Public Health, National University of Singapore and National University Health System, #09-01D, Tahir Foundation Building, 12 Science Drive 2, Singapore, 117549, Singapore, 65 65164988

**Keywords:** discrete choice experiment, nonpharmaceutical interventions, population preferences, endemic, COVID-19

## Abstract

**Background:**

Nonpharmaceutical interventions (NPIs) are effective tools for pandemic containment but often impose significant socioeconomic consequences that intensify over time. Public support and compliance to NPIs are crucial to ensure their effectiveness.

**Objectives:**

This study aimed to elicit preferences of a Singaporean population for the reintroduction of NPIs in response to the emergence of a new SARS-CoV-2 variant during the COVID-19 endemic phase.

**Methods:**

A web-based discrete choice experiment (DCE) was conducted. DCE attributes reflected key NPIs implemented in Singapore during the COVID-19 pandemic from 2020 to 2022, including mask wearing, dining restrictions, suspension of vocalization activities and large-scale events, quarantine after international travel, and mandatory vaccine boosters. Participants were recruited from a demographically representative online panel. Statistical analysis was performed using a mixed-logit model and mixed-mixed multinomial logit model.

**Results:**

A total of 1552 participants were included in the analysis. Overall preferences from the mixed-logit model showed that mask wearing was valued, both in public and indoors. Dining restrictions allowing groups of up to 5 people were preferred, but stricter dining restrictions allowing up to 2 people or no dining out were not favored. Prohibiting large-scale events was not preferred. Participants accepted quarantine at home but opposed quarantine in government facilities. Two classes emerged from the mixed-mixed logit model: class 1 (“Prefer NPIs”) and class 2 (“Prefer No NPIs”). While class 1 (39%) was only opposed to a complete prohibition on dining in at food and beverage establishments, no NPIs were preferred by class 2 (61%). Both classes were not opposed to mandatory mask wearing, dining restrictions allowing groups of up to 5 people, and mandatory vaccine boosters. Sex, age, education, employment status, the number of COVID-19 vaccine shots received, and risk attitude were associated with individuals’ likelihood of belonging to a specific preference group.

**Conclusions:**

Following the emergence of a new SARS-CoV-2 variant after a prolonged period of restrictions, less disruptive NPIs such as mask wearing indoors were valued by the public and should be swiftly reinstated. Adaptive strategies should be adopted for more contentious NPIs, such as strict dining restrictions and quarantine policies. Public preferences should be considered in the design and selection of NPIs for future pandemic containment strategies to enhance compliance and effectiveness.

## Introduction

### Background

Projections suggest that pandemics are likely to occur more frequently [1], with the annual probability of extreme epidemics potentially increasing 3-fold in the coming years due to factors such as climate change [2], zoonotic diseases [3], and increased cross-border travel [4]. As a result, pandemic preparedness is becoming increasingly crucial. Nonpharmaceutical interventions (NPIs) rose to prominence during the COVID-19 pandemic due to their central role in global containment strategies [5]. NPIs encompass all measures aimed at reducing or slowing disease transmission apart from vaccinations and medical interventions, such as mask wearing, social distancing, and movement restrictions [6,7]. The effectiveness of NPIs in reducing the transmissibility and severity of the SARS-CoV-2 virus [8-10], as well as decreasing the incidence of other infectious diseases [5,11-13], has been demonstrated in numerous studies. Nevertheless, the public health benefits of NPIs should be weighed against their broader societal impacts, as these measures also led to a host of socioeconomic consequences such as increased financial burden, mental health decline, and disrupted learning [14,15].

The success of NPIs hinges on public support and compliance, underscoring the need for strategies informed by population preferences [16]. Discrete choice experiments (DCEs) are widely used in public health to assess preferences by having participants choose among different scenarios, allowing researchers to analyze trade-offs and estimate willingness to pay (WTP) [17-20]. Several DCE studies have explored preferences for NPIs during the COVID-19 pandemic, primarily in Western populations [21-33], with 1 study in China [28]. These studies examined a range of attributes, from the health, social, and economic outcomes of NPIs to preferences for specific measures, such as mask mandates or stay home policies. However, most were conducted in the early phase of the pandemic, reflecting preferences during a period of heightened uncertainty.

Few studies have assessed population preferences for NPIs in the endemic phase of COVID-19, following a prolonged period of restrictions. As reported by Yang et al [28], respondents’ preferences for NPIs may change as a result of pandemic fatigue, a phenomenon characterized by declining adherence to restrictions over time [34,35]. Additionally, preferences for NPIs are shaped by demographic, socioeconomic, and cultural factors [16,34], limiting the generalizability of findings across regions. Understanding these influences is particularly crucial in an Asian context, where cultural and policy responses may differ from Western counterparts.

### Objectives

This study addresses these gaps by using a DCE to elicit Singaporeans’ preferences for NPIs in the event of a new SARS-CoV-2 variant during the COVID-19 endemic phase. By capturing preferences shaped by regional influences and real-world experiences with prolonged COVID-19 restrictions, the findings provide valuable insights for designing containment strategies that maximize public acceptance, minimize societal disruptions, and enhance long-term resilience against future health crises.

## Methods

### NPIs in Singapore

Following the detection of local COVID-19 clusters, safe distancing measures, including border restrictions and contact tracing, were introduced in Singapore in March 2020 [36]. By April, mask wearing in public became mandatory, and a “Circuit Breaker” was implemented, mandating the closure of all nonessential businesses, schools, and recreational facilities. Residents were instructed to stay home, with nonessential workers required to telecommute. Following the end of the Circuit Breaker in June 2020, restrictions on dining, group activities, and large-scale events were enforced. Large-scale events (eg, concerts and sporting events) and vocalization activities (eg, karaoke, choirs, and singing classes) were suspended, while limits on social gatherings and dining group sizes fluctuated based on outbreak severity. During times of heightened alert, dining in at food and beverage (F&B) establishments was completely suspended. Border controls remained in place throughout 2020 and 2021, requiring incoming travelers to quarantine either at government-designated facilities or at home. Extensive testing and contact-tracing efforts were adopted for the rapid identification and isolation of COVID-19 cases. Following the rollout of vaccination programs in late 2020, Vaccine-Differentiated Safe Management Measures and Vaccinated Travel Lanes were introduced in 2021. By early 2022, Singapore had transitioned to an endemic approach to COVID-19, with the easing of most NPIs in April 2022 marking a return to normalcy.

### DCE Question Design

Attributes were selected based on a review of key NPIs implemented in Singapore during the COVID-19 pandemic, as outlined in official public health guidelines [36-40]. The chosen NPIs were broad-based measures applicable to the entire population, rather than being specific to particular segments (eg, schools or workplaces). Attribute levels were derived from the actual tiers implemented during different phases of tightening and relaxation in Singapore, ensuring that they accurately reflected real-world policy shifts. Unlike conventional approaches that rely on literature reviews or stakeholder consultations, this method offers several advantages. As participants were familiar with the attributes and levels, ambiguity in scenario interpretation was reduced, allowing them to make choices based on lived experiences rather than purely hypothetical situations. Moreover, the alignment of study attributes with real-world NPIs ensures greater relevance for local policy makers and enhances policy impact. The selection of broad-based NPIs also strengthens the applicability of the study findings, as these measures have wider social and economic implications than segment-specific policies.

Table 1 outlines the seven attributes that were used in the DCE: (1) mandatory wearing of masks, (2) dining in group size restrictions, (3) restrictions on vocalization activities, (4) permission of large-scale events, (5) quarantine requirements after travel, (6) vaccine booster mandates, and (7) a one-time fee for public health measures. The inclusion of a cost attribute (one-time fee) is a standard approach in DCEs to estimate WTP, allowing for the assessment of how financial considerations influence public preferences for NPIs [41].

**Table 1.** Attributes and levels of the discrete choice experiment.

Attribute	Levels
Masks	Masks always required in public Masks required only when indoors No masks are required
Dining in	No dining in allowed Dining in for 2 people Dining in for groups of 5 Any group size can dine in
Vocalization activities	No vocalization activities allowed Vocalization activities allowed
Large-scale events	No large-scale events can be held Large-scale events can be held
Quarantine after travel	Quarantine in government facility Quarantine at home No quarantine
Vaccine booster	Compulsory Not compulsory
One-time fee (SGD $)^a^	$0 $10 $30 $50

a SGD $1≈US $0.77.

Sawtooth Software Lighthouse Studio (Academic Advanced Teaching Suite) was used to design the DCE questionnaire [42]. The balanced overlapping option was used to strike a balance between statistical efficiency and response efficiency. An unlabeled 1-stage design was used. The final design consisted of 20 blocks, each containing 7 DCE questions with 2 alternatives per question. Each attribute had up to 4 levels (Table 1). Participants were each assigned to 1 block of DCE questions at random.

The full survey consisted of 2 main parts: the first presented participants with 2 sets of DCEs, one with the above-mentioned design, and another from a different study design (not covered in this manuscript). The second part consisted of questions on sociodemographic information, including history of chronic diseases, COVID-19 vaccination status, employment, family background, housing type, teleconsultation use, and the Health Risk Attitude Scale (HRAS-13). The HRAS-13 is a 13-item scale designed to assess participants’ risk acceptance and tolerance in the health domain, with a higher sum score indicating a greater willingness to accept or tolerate potential health risks [43]. Its reliability and validity in the general population were established in a previous evaluation study [43]. The survey underwent 2 rounds of user acceptability testing with 15 and 20 staff members, respectively, to gather feedback on its suitability, clarity, and length. Based on this feedback, the survey refined before being disseminated to the full cohort. The complete set of survey instructions and a sample DCE question are shown in Multimedia Appendix 1.

### Sample, Sample Size, and Data Collection

Study participants were from the Singapore Population Health Studies (SPHS) Online Panel. This online platform facilitated public health research through monthly surveys on a diverse range of health-related topics. Participants eligible for the survey were Singapore citizens or permanent residents aged 18 years and older who met the SPHS Online Panel enrollment criteria, including the ability to read and comprehend English, and access to the web via a computer or a smartphone. Participants responded to the surveys hosted on the secure REDCap (Research Electronic Data Capture) system for National University of Singapore (NUS). At the time of survey dissemination, the panel had 2058 active participants who were demographically representative of the general population of Singapore. As participants were already enrolled in the SPHS Online Panel and had provided prior consent, no further recruitment was conducted. Participants could opt out of the questionnaire and choose not to participate in the study.

Using a rule of thumb formula n>500×*c*/(*t*×*a*) by Johnson and Orme [44], where 500 is a fixed variable, *c* demotes the largest number of levels for a certain attribute, *a* indicates the number of DCE choice sets per block of questionnaire, and *t* refers to the number of alternatives per DCE choice set, the sample size required for this study should be >143 participants (500×4/[2×7]=143). In addition, Lancsar and Louviere [45] had suggested 20 responses per block or questionnaire, thereby generating a minimum sample size of 400. Historically, the response rate for the SPH Online Panel Cohort has averaged about 78%, which would yield more than enough participants to identify the main effect [19,20].

### Ethical Considerations

The survey was administered from June 20, 2022, to June 29, 2022. SPHS Online Panel members were notified of the survey via email and text message on June 20, 2022, and were given 10 days to complete the survey. SPHS Online Panel was approved by the NUS Institutional Review Board (IRB; reference: H-18‐011). A waiver for documented informed consent for this study was granted by the NUS IRB (reference: NUS-IRB-2020‐82). Survey participation was entirely voluntary, and a participant information sheet was provided prior to starting the survey. An electronic consent procedure ensured that participants were informed of their rights before proceeding with the survey. Participants could withdraw from the study at any time without any obligation or penalty. To maintain confidentiality, all responses that the study team received were deidentified. Research data were securely stored in compliance with institutional data protection policies. Participants received SGD $15 (SGD $1≈US $0.77) as compensation upon survey completion.

### Variable Coding

To assess the impact of sociodemographic factors on preferences for NPIs, the study collected data on participants’ sex (male or female), ethnicity (Chinese or non-Chinese), marital status (married or not married), and parental status (with children or with no children). Education level (secondary school and below, or above secondary school and up to diploma equivalent, or university or higher) and housing type (private housing, such as condominiums, and larger government housing, such as 4- to 5-room housing development board flats, or smaller housing development board flats, such as 1- to 3-room units) were included as proxies for socioeconomic status [46,47]. Employment status was divided into 4 categories (full-time employed, self-employed or business owner, homemakers, others including part-time, unemployed, or retired). The number of COVID-19 vaccine shots received was grouped into 2 categories (zero to 2 shots or 3 shots and above), while age was treated as a continuous variable. HRAS-13 scores were also treated as a continuous variable, with higher values indicating less risk aversion.

### Statistical Analysis

Participants who refused or missed answering any of the survey questions were removed from the analysis. Two models were used in the analysis: mixed-logit (MXL) model and mixed-mixed multinomial logit (MMML) model [48]. The MXL model can account for random preference weight at individual level and relax the assumptions of independence of irrelevant alternatives [49]. Equation 1 shows the use for each individual, where $U_{ij}$ is the use for individual i when choosing choice j, $x_{j}$ is a vector describing choice j, $\beta_{i}$ is the preference weight or coefficient for individual i, and $\epsilon_{ij}$ is the idiosyncratic error term following a type 1 extreme value distribution.


(1) $$U_{ij}=\beta_{i}\times x_{j}+\epsilon_{ij}$$

Equation 2 shows the formula for $\beta_{i}$. β is the population mean. $\eta_{i}$ represents the random preference weight at the individual level following multivariate normal distribution, which includes all factors that affect individuals’ preference. Values for β are presented in the main results. SDs for $\eta_{i}$ are shown in Multimedia Appendix 2. Significant values for the SDs of $\eta_{i}$ means preference heterogeneity at individual level exists.


(2) $$\beta_{i}=\beta +\eta_{i}$$

The MMML model incorporates both the MXL model and the latent class model. The latent class model assumes that study participants can be assigned to unobserved (latent) classes based on their preference patterns. These preference weights are uniform within each class but vary systematically across classes. The MMML model works similarly by assigning study participants to unobserved classes. Mean preference weights, for example, β, are systematically different across classes. However, like the MXL model, people have same mean preference weights within each class but may have different preference weights at the individual level. Demographic information can be used to predict class membership. The optimal number of classes was chosen based on the Bayesian Information Criterion.

An alternative-specific constant (left) was included in each regression model, with a statistically significant coefficient indicating a left-right bias in the study [50]. Dummy coding was used for all attributes, with the exception of one-time fee for public health measures. For each attribute, the reference category was set as the lowest stringency of the NPI, that is, no restriction. Statistical significance was set at *P*<.05. All quantitative data analyses were carried out using R statistical software (version 4.2.2; The R Foundation) [51].

## Results

### Participant Characteristics

A total of 1651 participants were recruited in the study. After excluding participants with incomplete responses and 1 participant who indicated an age younger than 18 years, 1552 participants were included in the subsequent analysis. Participants had a mean age of 48 (SD 14) years, and a mean HRAS-13 score of 42 (SD 8). The remaining sociodemographic characteristics of the participants are summarized in Table 2. Compared with the demographic profile of the Singapore general population, the study sample had a higher median age and a higher proportion of females, individuals of Chinese ethnicity, and those with higher educational attainment (Multimedia Appendix 3).

**Table 2.** Distribution of sociodemographic characteristics of the study participants.

Characteristics	Frequency, n	Proportion, %
Total sample size	1552	N/A^a^
Sex		
Male	648	41.8
Female	904	58.2
Ethnicity		
Chinese	1314	84.7
Non-Chinese	238	15.3
Education		
Secondary school and below	335	21.6
Above secondary school and up to diploma equivalent	416	26.8
University and above	801	51.6
Housing		
1-3 room HDB^b^	343	22.1
4-5 room HDB^b^ or private property	1209	77.9
Marital status		
Married	1004	64.7
Unmarried	548	35.3
Parental status		
Have children	936	60.3
No children	616	39.7
Employment status		
Full-time	882	56.8
Self-employed or business owner	133	8.6
Homemaker	96	6.2
Others (eg, part-time, retired, or unemployed)	441	28.4
COVID-19 vaccine shots received		
0-2 shots	111	7.2
3 shots and above	1441	92.8

a N/A: not applicable.

b HDB: housing development board—government-provided housing.

### Regression Results

The regression results are shown in Tables3  4. Overall preferences of the study participants are described by the MXL model (Table 3). In the event of a new SARS-CoV-2 variant, mandatory mask wearing was valued, both in public (coefficient=0.54, 95% CI 0.44-0.64) and indoors (coefficient=0.59, 95% CI 0.49-0.69). Dining restrictions allowing groups of up to 5 were preferred (coefficient=0.19, 95% CI 0.08-0.30) while stricter dining restrictions of up to 2 people (coefficient=−0.33, 95% CI −0.44 to −0.22) or the complete prohibition of dining in at F&B establishments (coefficient=−0.89, 95% CI −1 to −0.78) was not favored. There was no significant preference regarding restrictions on vocalization activities (coefficient=−0.05, 95% CI −0.11 to 0.01); however, prohibiting large-scale events was not preferred (coefficient=−0.07, 95% CI −0.14 to 0). Participants were indifferent to home quarantine (coefficient=0.10, 95% CI 0-0.20) but opposed quarantine in government facilities (coefficient=−0.37, 95% CI −0.46 to −0.28). There was no clear preference for mandatory vaccine boosters (coefficient=0.03, 95% CI −0.03 to 0.09). Coefficients for unexplained preference heterogeneity at the individual level are shown in Multimedia Appendix 2. Overall, preference heterogeneity at the individual level was found for mandatory mask wearing (both public and indoors), complete prohibition of dining in at F&B establishments, quarantine at government facilities, and compulsory vaccine boosters.

**Table 3.** Mixed-logit model regression results.

	Coefficient	95% CI
Left	−0.08^a^	−0.14 to −0.02
Mask: Mandatory in public	0.54^b^	0.44 to 0.64
Mask: Mandatory indoors	0.59^b^	0.49 to 0.69
Dining in: Not allowed	−0.89^b^	−1 to −0.78
Dining in: Two people	−0.33^b^	−0.44 to −0.22
Dining in: Five people	0.19^b^	0.08 to 0.30
Vocalization activities: Not allowed	−0.05	−0.11 to 0.01
Large event: Not allowed	−0.07	−0.14 to 0
Quarantine: Government facility	−0.37^b^	−0.46 to−0.28
Quarantine: Home	0.10	0 to 0.20
Vaccine booster: Mandatory	0.03	−0.03 to 0.09
Fee for public health measure	−0.02^b^	−0.02 to −0.02

a *P*<.05.

b *P*<.001.

**Table 4.** Mixed-mixed multinomial logit model regression results.

	Class 1: Prefer NPI^a^ (39%)			Class 2: Prefer No NPI (61%)		
	Coefficient	95% CI	*P* value	Coefficient	95% CI	*P* value
Left	−0.19	−0.34 to −0.04	.01	0.11	0.01 to 0.21	.04
Mask: Mandatory in public	1.98	1.63 to 2.33	<.001	−0.37	−0.53 to −0.21	<.001
Mask: Mandatory indoors	1.76	1.43 to 2.09	<.001	0.04	−0.11 to 0.19	.62
Dining in: Not allowed	−0.51	−0.75 to −0.27	<.001	−1.72	−1.98 to −1.46	<.001
Dining in: Two people	0.18	−0.09 to 0.45	.20	−0.87	−1.07 to −0.67	<.001
Dining in: Five people	0.49	0.21 to 0.77	<.001	−0.08	−0.26 to 0.10	.36
Vocalization activities: Not allowed	−0.06	−0.2 to 0.08	.42	−0.12	−0.22 to ‐0.02	.02
Large event: Not allowed	0.21	0.04 to 0.38	.01	−0.29	−0.41 to −0.17	<.001
Quarantine: Government facility	0.35	0.13 to 0.57	.002	−1.01	−1.19 to −0.83	<.001
Quarantine: Home	0.56	0.31 to 0.81	<.001	−0.19	−0.35 to −0.03	.02
Vaccine booster: Mandatory	−0.02	−0.17 to 0.13	.80	−0.02	−0.12 to 0.08	.64
Fee for public health measure	−0.02	−0.03 to −0.01	<.001	−0.04	−0.05 to −0.03	<.001

a NPI: nonpharmaceutical intervention.

In the MMML model, participants were grouped into 2 classes (Table 4). Class 1 was defined as “Prefer NPI,” making up 39% of the sample, while class 2 was defined as “Prefer No NPI,” comprising the remaining 61%. Class 1 was only opposed to prohibition on dining in at F&B establishments (coefficient=−0.51, 95% CI −0.75 to −0.27). Mandatory mask wearing was highly valued, both in public (coefficient=1.98, 95% CI 1.63-2.33) and indoors (coefficient=1.76, 95% CI 1.43-2.09). For class 2, none of the NPIs were preferred. However, they were not averse to mandatory mask wearing indoors (coefficient=0.04, 95% CI −0.11 to 0.19), dining restrictions allowing groups of up to 5 people (coefficient=−0.08, 95% CI −0.26 to 0.10), and mandatory vaccine booster (coefficient=−0.02, 95% CI −0.12 to 0.08). Coefficients for unexplained preference heterogeneity at the individual level are summarized in Multimedia Appendix 2.

**Figure 1.**
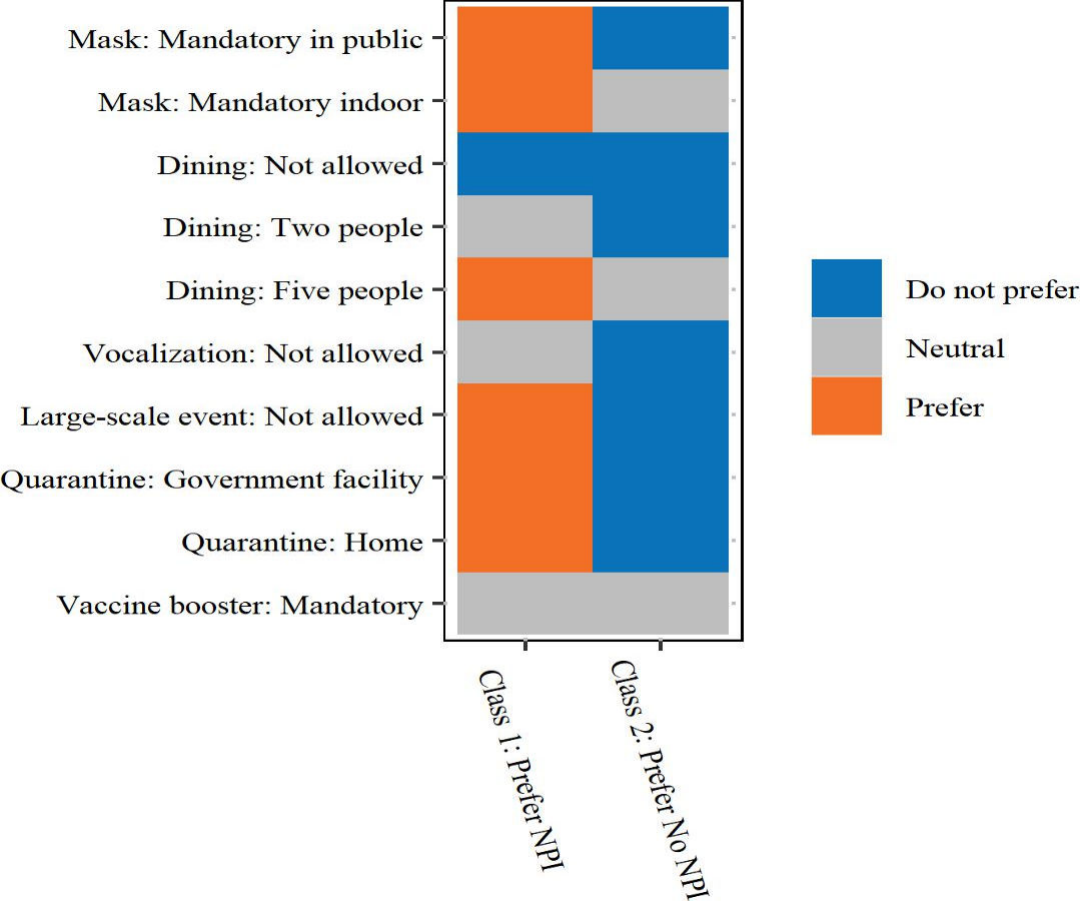
Preferences for NPIs for class 1 (Prefer NPI) and class 2 (Prefer No NPI). NPI: nonpharmaceutical intervention.

Figure 1 provides an overview of participants’ preferences for the given NPIs. Neither of the classes were averse to mandatory mask wearing indoors, dining restrictions allowing groups of up to 5 people, and mandatory vaccine boosters. However, both groups were opposed to a complete prohibition on dining in at F&B establishments.

**Figure 2.**
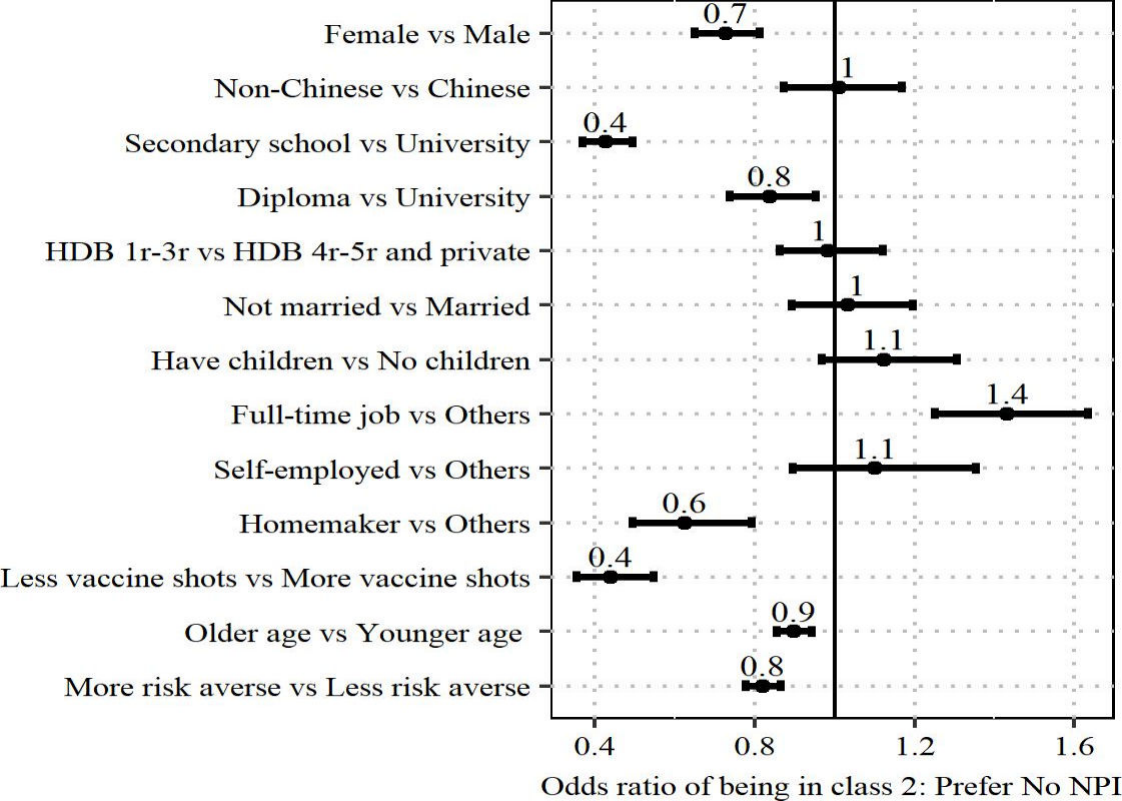
Odds ratio of being in class 2 (Prefer No NPI) comparing different demographic groups. An odds ratio greater than 1 indicates a higher likelihood of being in class 2 for the first group listed, while an odds ratio less than 1 indicates a lower likelihood of being in class 2 for the first group compared with the second group (eg, female vs male). HDB: housing development board; NPI: nonpharmaceutical intervention.

Figure 2 shows the odds ratio for membership in class 2 (Prefer No NPI). Men were more likely to belong in class 2 and prefer no NPIs as compared with women. Higher education levels were also associated with higher odds of preferring no NPIs. Those in full-time employment preferred no NPIs; in contrast, homemakers were more likely to prefer NPIs. Respondents who had received fewer vaccine shots tended to prefer NPIs, compared with those who had received more. Additionally, older age and higher risk aversion were associated with higher odds of preferring NPIs.

### Willingness to Pay

**Figure 3.**
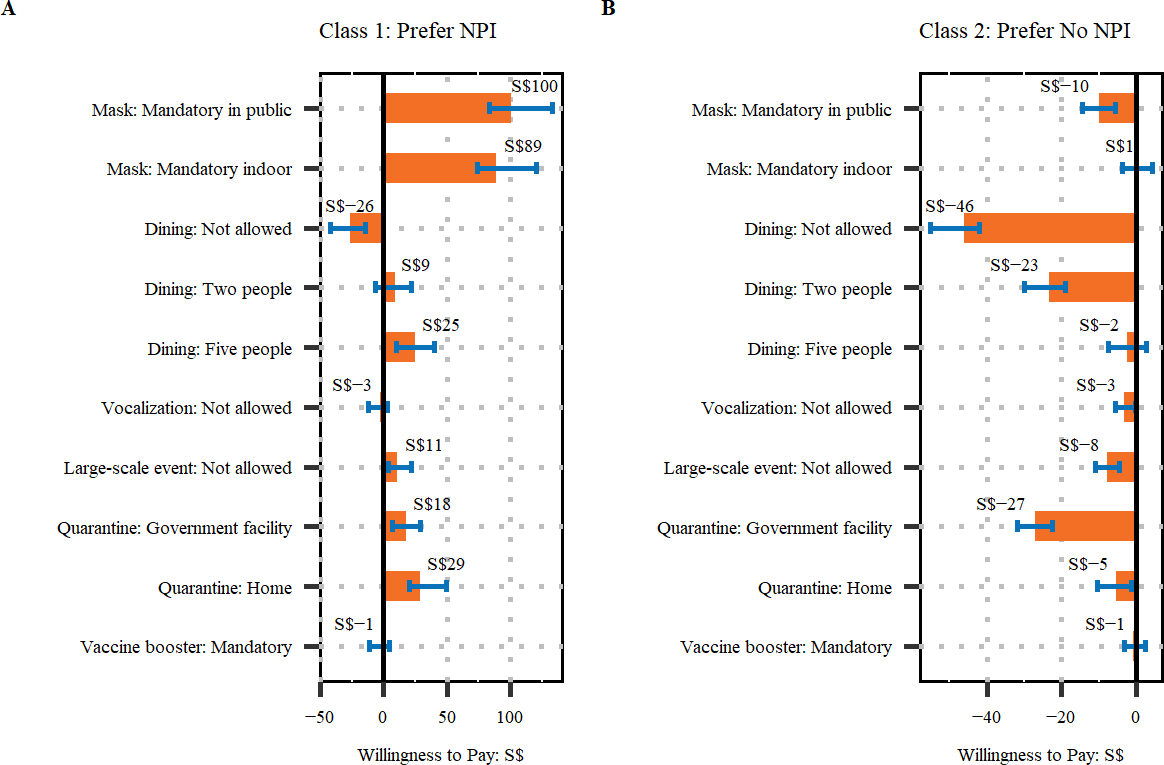
Willingness to pay (WTP) for each nonpharmaceutical intervention (NPI) for (A) class 1 (Prefer NPI) and (B) class 2 (Prefer No NPI). WTP reflects the amount that participants are willing to pay for the implementation of the NPI. A positive WTP indicates that participants were willing to pay to implement the NPI. A negative WTP indicates that participants were willing to pay to avoid the NPI. S$: Singapore dollar.

Willingness to pay, measured by the fee for implementing public health measures, is shown in Figure 3A-B. For participants in class 1 (Prefer NPI) as shown in Figure 3A, the top 3 measures they were willing to pay for were mandatory mask wearing in public (SGD $100; SGD $1≈US $0.77), mandatory mask wearing indoors (SGD $89), and home quarantine (SGD $29). They were also willing to pay SGD $26 to avoid a complete prohibition on dining in at F&B establishments. For people in class 2 (Prefer No NPI) as shown in Figure 3B, the top 3 NPIs they were willing to pay to avoid were a complete prohibition on dining in at F&B establishments (SGD $46), quarantine at government facilities (SGD $27), and dining group size restrictions of up to 2 people (SGD $23).

## Discussion

### Principal Findings

This study provides valuable insights into public preferences for NPIs during the endemic phase of COVID-19, particularly in the face of a new viral variant. The findings build on existing research, including Lim et al [52], which examined public perceptions and behaviors toward NPIs in Singapore during the early stages of the pandemic through cohort surveys. This study extends these insights by exploring NPI acceptability in a later phase of the pandemic, when pandemic fatigue may have influenced public attitudes. Overall, mandatory mask wearing in public and indoor settings was well received, while strict dining restrictions, quarantine in government facilities, and the suspension of large-scale events faced opposition. A latent class analysis revealed 2 distinct preference groups: those who preferred NPIs (Prefer NPI; 39%), and those who opposed them (Prefer No NPI; 61%). The Prefer NPI group strongly supported mask mandates and only opposed a complete prohibition on dining in at F&B establishments, whereas the Prefer No NPI group exhibited no preference for any NPIs but was not averse to indoor masking and moderate dining restrictions. NPI preferences were influenced by demographic factors such as age, sex, education, employment status, COVID-19 vaccination history, and risk aversion.

A prohibition on dining in at F&B establishments emerged as the least favored NPI among both classes and was the only NPI that faced opposition in class 1 (Prefer NPI). This aligns with the findings by Fink et al [30] on the closure of restaurants and bars, highlighting the social and cultural significance of dining in at such establishments. Singapore’s UNESCO-recognized hawker centers, often referred to as “community dining rooms,” are central to both social and recreational life [53]. Therefore, strict dining restrictions in regions with strong dining out cultures have proven to be particularly disruptive and unacceptable, especially when imposed for prolonged periods.

Moreover, the suspension of large-scale events was not favored in this study, consistent with the strong opposition reported by Fink et al [30] toward restrictions on social events such as parties, weddings, and concerts. Similarly, Lim et al [52] reported relatively low public support for bans on social gatherings and penalties for noncompliance with social distancing measures, suggesting public resistance to stringent restrictions on social events in Singapore. The similarity in findings suggests that public resistance to the suspension of large-scale events and gatherings in Singapore has remained relatively constant throughout the pandemic, even as different phases of COVID-19 unfolded. In contrast, Eshun-Wilson et al [31] identified the suspension of large gatherings as the most favored NPI. Differences in preferences could be attributed to the role of social and cultural norms, as such restrictions may be more disruptive in regions with strong social and communal event cultures.

Comparatively, mandatory mask wearing, a widely adopted NPI during the pandemic, garnered strong public support across the literature, including in this study [29,32,33]. This finding aligns with the results from Lim et al [52], where 99.0% of respondents reported wearing masks most or all of the time, even before the official implementation of the mask mandate in Singapore. The widespread support for mandatory mask wearing underscores its recognition as an effective, relatively nondisruptive intervention, which could be easily reintroduced in the event of new epidemic waves. Additionally, Singapore’s prior experience with the SARS outbreak likely played a role in fostering this high level of compliance. During SARS, prior mask usage was found to be a positive predictor of support for mask wearing, suggesting that familiarity with such measures contributed to greater public adherence [54]. This historical precedent may have contributed to the high acceptance observed in this study, as Singaporeans were already accustomed to wearing masks as a protective measure.

In addition, this study examined public preferences for a vaccine booster mandate. In contrast to the findings by Yang et al [28], which indicated a preference for universal vaccine boosters among Chinese respondents, respondents in this study exhibited indifference toward a vaccine booster mandate. This divergence in public perceptions reflects the distinct COVID-19 strategies used by each country. At the time of data collection for both studies (mid-2022), China continued to enforce a strict zero-COVID policy, while Singapore had transitioned to an endemic phase of living with COVID-19. This could potentially contribute to a lower perceived need for booster doses among Singaporeans, despite the emergence of new COVID-19 variants.

Overall support for NPIs varied significantly across different demographic groups, particularly by vaccination and employment status. Participants who had received a higher number of vaccine doses generally showed a preference for no NPIs, even in the face of new virus variants. The added protection from additional vaccine doses, combined with vaccine passports that granted vaccinated individuals with greater freedom, may have contributed to a perception of lower susceptibility to new COVID-19 variants. Consequently, this group exhibited a lower perceived need and preference for NPIs. Employment status also played a crucial role in shaping NPI preferences, as full-time employees in Singapore were less likely to support NPIs. This contrasts with the findings by Reed et al [27] in the United States, where salaried employees preferred more stringent NPIs than self-employed individuals. This disparity could be attributed to differences in economic structures, as Singapore had a higher reliance on physical workplaces than the United States, where remote work was more widely adopted. Concerns about job and financial security in Singapore were evident in public discourse even in the early phases of the pandemic, where many voiced anxieties over the potential consequences of the circuit breaker regulations on job stability and work-life balance [55]. To enhance public acceptance of NPIs in future outbreaks, policy makers could consider strategies that alleviate financial strain—such as targeted financial relief and flexible enforcement policies—to balance public health objectives with economic stability.

Beyond these demographic influences, unexplained individual preference heterogeneity was observed in both regression models, particularly for mandatory mask wearing, prohibition on dining in at F&B establishments, quarantine in government facilities, and vaccine booster mandates. This suggests that unobservable personal, experiential, or psychological factors may influence individuals’ views on NPIs, underscoring the challenge of designing public health policies that accommodate these diverse preferences. Future studies can explore the factors affecting the preferences for NPIs that exhibit high degree of heterogeneities at an individual level. Adaptive strategies, such as tiered restrictions based on public sentiment and risk assessment, are crucial in maximizing policy acceptability and compliance. This is especially relevant for contentious measures such as strict dining restrictions and quarantine policies, where strong opposition may lead to rapid declines in adherence and effectiveness [56].

### Strengths and Limitations

One strength of this study is its exploration of public preferences for less commonly studied NPIs, such as quarantine after travel and dining group size restrictions. These insights can help inform their design and implementation in future pandemic containment strategies in Singapore and beyond. Additionally, the use of actual COVID-19 policy measures implemented in Singapore between 2020 and 2022 as DCE attributes allowed participants to draw from personal experiences when making decisions. This reduced hypothetical bias and led to more informed responses that better reflect real-world preferences [57]. However, since the survey was conducted soon after the lifting of most pandemic restrictions in Singapore, responses may have been influenced by recency bias, with participants forming stronger opinions based on their recent experiences with the NPIs. Furthermore, preferences for NPIs are likely shaped by participants’ expectations regarding the severity of a new variant at the time of the survey. Future studies could further explore how perceptions of disease severity influence public support for NPIs.

The generalizability of the study findings to the broader Singapore population may be limited. Participants recruited from the SPHS Online Panel had high English proficiency and digital literacy, potentially excluding individuals with lower proficiency or limited access to digital devices—more common among older adults. However, Singapore’s high overall English literacy and internet penetration rates mitigate some of these concerns [58,59]. Nevertheless, some of the demographic differences that exist between the study sample and the general population may still affect generalizability, since demographic factors were shown to influence NPI preferences. Future studies could use multilingual surveys to explore preferences among non–English-speaking older adults and ensure a more representative sample. Beyond demographics, public acceptance of NPIs is also shaped by broader cultural and geographic contexts, as evidenced by significant regional differences in NPI support during the SARS outbreak [54]. This underscores the importance of flexible, context-specific public health strategies rather than a one-size-fits-all approach. Given these factors, findings from this study may be more applicable to high-income Asian settings, where similar characteristics such as dense urban environments, strong institutional trust, and prior epidemic experience may shape NPI preferences differently from Western or lower-income settings.

### Implications and Conclusions

The findings from this study are particularly relevant as the world continues to navigate the shifting landscape of infectious disease outbreaks. In the endemic phase of COVID-19 and other infectious diseases, the emergence of new viral variants may necessitate the reintroduction of NPIs as complementary tools to control outbreaks before the widespread uptake of vaccine boosters. A thorough understanding of public preferences is essential for designing policies that not only facilitate the reimplementation of NPIs following new viral outbreaks but also determine their optimal duration to ensure compliance and effectiveness while minimizing societal disruptions. NPIs with broad support, such as indoor mask wearing, can be swiftly reinstated and maintained over extended periods with minimal resistance. However, for NPIs that elicit mixed preferences or strong opposition—such as strict dining restrictions and quarantine in government facilities—a careful cost-benefit analysis is necessary. The use of adaptive strategies, such as phased implementation or targeted enforcement based on risk levels, may help balance public health objectives with societal acceptance. By aligning pandemic containment measures with public sentiment, policy makers can enhance adherence, minimize disruptions, and strengthen long-term resilience against future disease outbreaks.

## Supplementary material

10.2196/67725 Multimedia Appendix 1 Discrete choice experiment scenario and sample question.

10.2196/67725 Multimedia Appendix 2 Unexplained individual preference heterogeneity.

10.2196/67725 Multimedia Appendix 3 Comparison of demographic profile between the sample and Singapore population.

## Abbreviations

DCE: discrete choice experiment
F&B: food and beverage
HRAS-13: Health Risk Attitude Scale
IRB: institutional review board
MMML: mixed-mixed multinomial logit
MXL: mixed-logit
NPI: nonpharmaceutical intervention
NUS: National University of Singapore
REDCap: Research Electronic Data Capture
SPHS: Singapore Population Health Studies
WTP: willingness to pay
