# Supplementary material for: Preferences for Nonpharmaceutical Interventions During the Endemic Phase of COVID-19: Discrete Choice Experiment
Source: JMIR Public Health Surveill. 2025 Jun 4;11:e67725. doi: 10.2196/67725 (PMC12157962; doi:10.2196/67725)
Supplement: Multimedia Appendix 1 [file publichealth-v11-e67725-s001.docx]

**DCE Survey Instructions and Sample Question**

DCE Survey Instructions

For this part of the survey, we want you to imagine a possible situation next year when there is a new COVID-19 variant.

In this imaginary situation, there might be different combinations of safe management measures that could be put in place in Singapore, including possible compulsory vaccine booster to protect against this new COVID-19 variant.

The vaccine booster is 90% effective in preventing getting infected against this new variant for 9 months.

The vaccine booster has a safety profile of about 0.1% risk of adverse events (1 in 1,000 experience effects such as dizziness, chest tightness, pain and swelling of injection site, or fever), and about 0.005% risk of severe adverse events (5 in 100,000 experience severe effects such as anaphylaxis [severe allergic reaction], myocarditis [heart inflammation], or other life-threatening reactions).

There will also be different amounts that you might have to pay as a one-time fee for public health measures.

You will be asked to choose which one of the two scenario options you prefer.

Here is an example of a possible set of options you will see in the following set of 7 questions:

**Between the two scenario options next year, which would you prefer?**

|  | Option 1 |  | Option 2 |
| --- | --- | --- | --- |
| **Masks** | Masks always required in public |  | No masks are required |
| **Dining in** | Any group size can dine in |  | Dining in for 2 people |
| **Vocalisation activities** | No vocalisation activities allowed |  | Vocalisation activities allowed |
| **Large-scale events** | Large-scale events can be held |  | No large-scale events can be held |
| **Quarantine after travel** | No quarantine |  | Quarantine at home |
| **Vaccine booster** | Not compulsory |  | Compulsory |
| **One-time fee** | $30 |  | $0 |

Please read through the following carefully to understand the terms used:

- **Masks** refer to face masks that will be worn cover the nose and the mouth.
- **Dining in** refers to consuming food and beverage (F&B) in establishments such as restaurants, fast food outlets, cafes, food courts, etc. There can be limits on the number of people that can dine together.
- **Vocalisation activities** refer to activities such as karaoke, cheering/singing at weddings, and chanting/prayers/singing at religious events.
- **Large-scale events** refer to activities that would have large groups of people attending for a long period of time, such as concerts, sports events, and festivals.
- **Quarantine after travel** refers to whether you have to be quarantined after returning to Singapore from travelling overseas. Quarantine could be in a dedicated government facility, or it could be in one’s home.
- **One-time fee** refers to the amount of money that you have to pay out of pocket to fund public health measures.

Sample DCE question


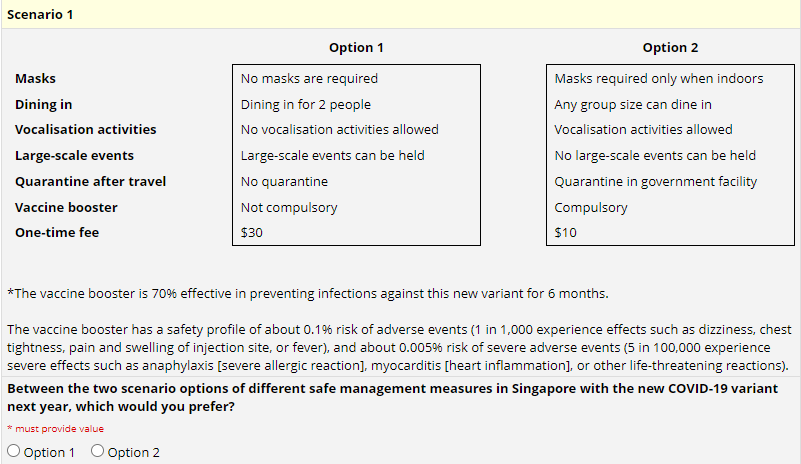


Figure 1. Screenshot of a sample DCE question presented to participants.
