# Supplementary material for: Preferences for Nonpharmaceutical Interventions During the Endemic Phase of COVID-19: Discrete Choice Experiment
Source: JMIR Public Health Surveill. 2025 Jun 4;11:e67725. doi: 10.2196/67725 (PMC12157962; doi:10.2196/67725)
Supplement: Multimedia Appendix 2 [file publichealth-v11-e67725-s002.docx]

Table 1. Coefficients for Unexplained Individual Preference Heterogeneity – Standard Deviation of Coefficients.

|  | Mixed-logit Model | | Mixed-mixed-logit Model | | | |
| --- | --- | --- | --- | --- | --- | --- |
|  |  |  | **Class 1: Prefer NPI**^c^ **(39%)** | | **Class 2: Prefer No NPI**^c^ **(61%)** | |
|  | **SD^a^ of Coefficient** | **95% CI^b^** | **SD^a^ of Coefficient** | **95% CI^b^** | **SD^a^ of Coefficient** | **95% CI^b^** |
|  |  |  |  |  |  |  |
| Left | 0.46 | (0.31, 0.61) | 0.35 | (-0.08, 0.78) | 0.75 | (0.53, 0.97) |
| Mask: Mandatory in Public | 1.05 | (0.89, 1.21) | 0.97 | (0.58, 1.36) | 0.63 | (0.31, 0.95) |
| Mask: Mandatory Indoor | 0.32 | (0.05, 0.59) | 0.8 | (0.36, 1.24) | 0.20 | (-0.27, 0.67) |
| Dining: Not Allowed | 0.85 | (0.64, 1.06) | 0.45 | (-0.12, 1.02) | 1.39 | (1.03, 1.75) |
| Dining: Two People | 0.04 | (-0.31, 0.39) | 0.41 | (-0.23, 1.05) | 0.11 | (-0.38, 0.6) |
| Dining: Five People | 0.13 | (-0.2, 0.46) | 0.64 | (0.07, 1.21) | 0.41 | (-0.02, 0.84) |
| Vocalisation: Not Allowed | 0.19 | (0, 0.38) | 0.58 | (0.27, 0.89) | 0.31 | (0.04, 0.58) |
| Large Event: Not Allowed | 0.16 | (-0.06, 0.38) | 0.37 | (-0.06, 0.8) | 0.34 | (0.04, 0.64) |
| Quarantine: Government Facility | 0.60 | (0.42, 0.78) | 0.19 | (-0.23, 0.61) | 0.68 | (0.37, 0.99) |
| Quarantine: Home | 0.03 | (-0.2, 0.26) | 0.46 | (0.09, 0.83) | 0.05 | (-0.33, 0.43) |
| Vaccine Booster: Mandatory | 0.95 | (0.83, 1.07) | 2.10 | (1.72, 2.48) | 0.37 | (0.14, 0.6) |
| Fee for public health measure | 0.02 | (0.02, 0.02) | 0.01 | (0, 0.02) | 0.04 | (0.03, 0.05) |

^a^SD: standard deviation

^b^CI: confidence interval

^c^NPI: non-pharmaceutical intervention
