# Supplementary material for: Preferences for Nonpharmaceutical Interventions During the Endemic Phase of COVID-19: Discrete Choice Experiment
Source: JMIR Public Health Surveill. 2025 Jun 4;11:e67725. doi: 10.2196/67725 (PMC12157962; doi:10.2196/67725)
Supplement: Multimedia Appendix 3 [file publichealth-v11-e67725-s003.docx]

Table 1. Comparison of Demographic Profile Between DCE Sample and Singapore Population

| Source | |  | | Current study | Census of Population 2020 [1] | |
| --- | --- | --- | --- | --- | --- | --- |
|  | |  | |  | |  |
| **Total sample/population size** | | | | 1,552 | | 4,044,210 |
| **Gender** | | |  |  |  |  |
|  | Male | | | 41.8% | | 48.9% |
|  | Female | | | 58.2% | | 51.1% |
| **Ethnicity** | | |  |  |  |  |
|  | Chinese | | | 84.7% | | 74.3% |
|  | Malay, Indian, or Others | | | 15.3% | | 26.7% |
| **Education** | | |  |  |  |  |
|  | Secondary school and below | | | 21.6% | | 26.7% |
|  | Above secondary school and up to diploma equivalent | | | 26.8% | | 41.8% |
|  | University and above | | | 51.6% | | 25.3% |
| **Marital Status** | | |  |  |  |  |
|  | Married | | | 64.7% | | 58.8% |
|  | Single, Widowed, or Divorced/Separated | | | 35.3% | | 41.1% |
|  |  | | |  | |  |
|  | Age (median) | | | 48 | | 41.2 |
